# Systemic administration of clinical-grade multilineage-differentiating stress-enduring cells ameliorates hypoxic–ischemic brain injury in neonatal rats

**DOI:** 10.1038/s41598-023-41026-3

**Published:** 2023-09-11

**Authors:** Kazuto Ueda, Yoshiaki Sato, Shinobu Shimizu, Toshihiko Suzuki, Atsuto Onoda, Ryosuke Miura, Shoji Go, Haruka Mimatsu, Yuma Kitase, Yuta Yamashita, Keiichi Irie, Masahiro Tsuji, Kenichi Mishima, Masaaki Mizuno, Yoshiyuki Takahashi, Mari Dezawa, Masahiro Hayakawa

**Affiliations:** 1 Division of Neonatology, Center for Maternal-Neonatal Care, Nagoya University Hospital, 65 Tsurumai-cho Showa-ku, Nagoya, 466-8560 Japan; 2 Department of Pediatrics, Nagoya University Graduate School of Medicine, Nagoya, Japan; 3 Department of Advanced Medicine, Nagoya University Hospital, Nagoya, Japan; 4 Faculty of Pharmaceutical Sciences, Sanyo-Onoda City University, Yamaguchi, Japan; 5 Department of Pharmacology, Faculty of Pharmaceutical Sciences, Fukuoka University, Fukuoka, Japan; 6 Department of Food and Nutrition, Faculty of Home Economics, Kyoto Women’s University, Kyoto, Japan; 7 Department of Stem Cell Biology and Histology, Tohoku University Graduate School of Medicine, Sendai, Japan

**Keywords:** Stem cells, Neurology

## Abstract

Multilineage-differentiating stress-enduring (Muse) cells are endogenous reparative pluripotent stem cells present in the bone marrow, peripheral blood, and organ connective tissues. We assessed the homing and therapeutic effects of systemically administered nafimestrocel, a clinical-grade human Muse cell-based product, without immunosuppressants in a neonatal hypoxic–ischemic (HI) rat model. HI injury was induced on postnatal day 7 (P7) and was confirmed by T2-weighted magnetic resonance imaging on P10. HI rats received a single dose nafimestrocel (1 × 10^6^ cells/body) or Hank’s balanced salt solution (vehicle group) intravenously at either three days (on P10; M3 group) or seven days (on P14; M7 group) after HI insult. Radioisotope experiment demonstrated the homing of chromium-51-labeled nafimestrocel to the both cerebral hemispheres. The cylinder test (M3 and M7 groups) and open-field test (M7 group) showed significant amelioration of paralysis and hyperactivity at five weeks of age compared with those in the vehicle group. Nafimestrocel did not cause adverse events such as death or pathological changes in the lung at ten weeks in the both groups. Nafimestrocel attenuated the production of tumor necrosis factor-α and inducible nitric oxide synthase from activated cultured microglia in vitro. These results demonstrate the potential therapeutic benefits and safety of nafimestrocel.

## Introduction

Neonatal hypoxic–ischemic encephalopathy (HIE), one of the most severe neurological diseases during perinatal period^1,2^, causes permanent neurological deficits or neonatal death^3^. There are still no effective treatments available for HIE except hypothermia^4^. Clinical trials for hypothermia demonstrate a decreased risk of death and neurological deficits in newborns with HIE; however, such beneficial effects were limited: the number needed to treat was 9 (95% CI 5 to 25) for hypothermia therapy to avoid one death or severe disability at 18 months^5^. Therefore, exploration of novel treatment is one of the key tasks in clinical research of HIE.

Stem cell therapy has been developed as one of the promising treatments for central nervous system diseases^6–8^. Various kinds of stem cells such as mesenchymal stem cells (MSCs) have been assessed for clinical applications^9–13^.

Multilineage-differentiating stress-enduring (Muse) cells are endogenous, nontumorigenic, pluripotent stem cells, which can be collected as cells positive for pluripotent surface marker, stage specific embryonic antigen (SSEA)-3^14–17^. Muse cells are found in the bone marrow, peripheral blood and connective tissues of various organs^18^. In addition to their non-tumorigenicity, triploblastic differentiation ability, self-renewability, and stress tolerance, Muse cells possess several unique features that make them highly practical for cell therapy; 1) surgical procedures are not required for target organ delivery, since they express sphingosine-1-phosphate (S1P) receptor 2, enabling them to selectively home to the damage site after intravenous injection by sensing general damage signal, S1P^15,19^.; 2) gene introduction or differentiation induction is not necessary prior to their administration because Muse cells differentiate spontaneously into multiple cell types that constitute the tissue and replace damaged/apoptotic cells, leading to tissue repair^20–28^.; 3) human leukocyte antigen matching or immunosuppressants are not required for the use of donor-derived Muse cells due to their immune privilege system, partly explained by the expression of HLA-G^19^, relevant to immunosuppression in the placenta^29^ and by the production of interferon gamma-induced indoleamine-2,3 dioxygenase^20^. Indeed, allogeneic Muse cells escaped immune rejection and survived as functional cells in the host tissue for over 6 months without immunosuppressants^19^. Currently, clinical trials for stroke, acute myocardial infarction, epidermolysis bullosa, spinal cord injury, amyotrophic lateral sclerosis, and acute respiratory distress syndrome with COVID-19 are conducted by intravenous injection of clinical-grade human Muse cell-based product, CL2020 (the cells in CL2020 were named nafimestrocel as international non-proprietary name), without HLA matching and immunosuppressants under the permission of regulatory authority (Japan Pharmaceutical Information Center-Clinical Trials Information; JapicCTI-183834, JapicCTI-184103, JapicCTI-184563, JapicCTI-194841, JapicCTI-195067, jRCT2063200047, and jRCT2043210005). The safety and effectiveness of CL2020 is reported in acute myocardial infarction^30^ and in epidermolysis bullosa^31^.

We previously reported potential therapeutic effects of fluorescence-activated cell sorting (FACS)-isolated research-grade human Muse cells on HIE in a rat model^32^. Human bone marrow-derived Muse cells were administered intravenously at three days after insult, which homed to the ischemic region, differentiated spontaneously into neural cells in the homed brain tissue, and significantly improved brain function at 5 months in the absence of immunosuppressants. Glutamate metabolism was modulated, and microglial activation was alleviated by Muse cells^32^. These findings strongly suggest that administration of Muse cells is a novel therapeutic approach against neonatal HIE.

However, in order to translate these results into clinical practice, the corresponding benefits, the timing of treatment, and the feasibility of using nafimestrocel without using any immunosuppressive agent need to be verified. In the present study, we investigated how the homing and therapeutic effect will be affected by the timing of nafimestrocel administration in a rat model of hypoxic–ischemic (HI)injury. We also evaluated the effects of nafimestrocel on activated cultured microglia in vitro.

## Results

A total of 33 male Wistar/ST rat pups including three sham rats were used in this study. Based on clinical relevance (described in Methods), the mildly or severely injured animals evaluated by magnetic resonance imaging were excluded. 30 moderately injured rats were only used in subsequent experiments. Of these, 27 were used for behavioral and histological evaluation. For the radioisotope experiment, six rats were used, comprising of three moderately injured rats and three sham rats. HI insult was induced on postnatal day 7 (P7).

### In vivo dynamics of intravenously administered nafimestrocel by using radioisotopes

Radiolabeling and tracking with chromium-51 radionuclide was used to examine cell homing, based on the previously reported method with modifications^33–35^. Briefly, the severity of brain injury was evaluated by diffusion-weighted MRI 3–5 h after HI insult, and three moderately injured rats were adopted. A single dose ^51^Cr-labeled nafimestrocel (1 × 10^6^ cells/body) was administered intravenously for three HI rats and three sham rats on P10. Brain tissue was collected 24, 72 and 168 h after administration, and the frozen-sections were prepared. Each section was used to obtain radioluminograms. The homing of nafimestrocel was calculated as radioactivity concentration using radioluminograms.

### In vivo dynamics of ^51^Cr-labeled nafimestrocel

**Figure 1:**
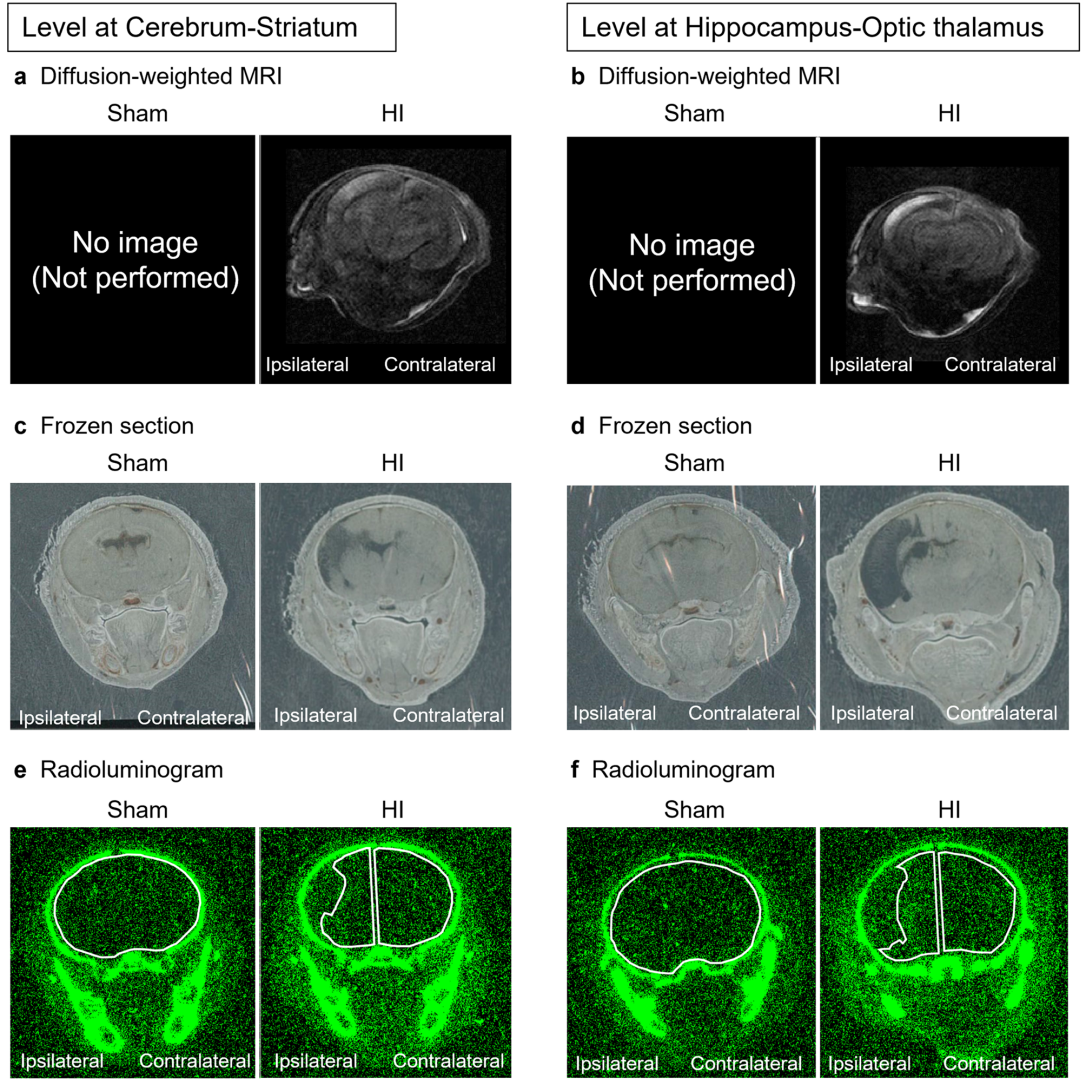
Representative images of diffusion-weighted magnetic resonance imaging (MRI; **a**, **b**), histological frozen sections (**c**, **d**) and radioluminograms (**e**, **f**). Diffusion-weighted MRI was performed prior to treatment, and frozen sections and radioluminograms were prepared 72 h after a single intravenous administration of [^51^Cr]-nafimestrocel. Each region of interest (ROI) in the radioluminograms is indicated with a white outlined area.

**Figure 2:**
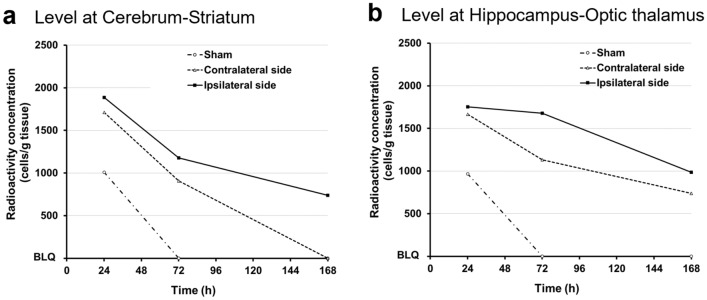
Radioactivity concentration in the brain after a single intravenous administration of [^51^Cr]-nafimestrocel. (**a**) Level at cerebrum-striatum. (**b**) Level at hippocampus-optic thalamus.* n* = 1 at each time for the sham group (*n* = 3), the ipsilateral (left) side in the hypoxic–ischemic (HI) group (*n* = 3), and the contralateral side in the HI group (*n* = 3).

Representative images of diffusion-weighted MRI and frozen-section histological analysis are shown in Fig. 1a–d, respectively. In HI rats, HI insult resulted in tissue collapse mainly in the cortex and a part of the hippocampus. Representative radioluminograms are shown in Fig. 1e and f. In HI rats, ROIs were set along the periphery of remaining parenchyma on the ipsilateral (left) and contralateral sides, as HI insult led to a partial loss of brain parenchyma (Fig. 1c, d, e, f). Radioactivity concentration from 24 to 168 h after ^51^Cr-labeled nafimestrocel administration is shown in Fig. 2a and b. Radioactivity concentration in each cerebral hemisphere of HI rats was higher than that in the sham group, and that in both the cerebrum-striatum and hippocampus-optic thalamus levels on the ipsilateral side of HI rats was higher than that on the contralateral side from 24 to 168 h after ^51^Cr-labeled nafimestrocel administration (Fig. 2a, b).

### Safety assessment and behavioral experiments

For safety assessment and behavioral experiments, moderately injured rats assessed by T2-weighted MRI on P10 were used. Systemic administration of nafimestrocel (1 × 10^6^ cells/body) was performed on either P10 (day 3 after HI; the M3 group) or P14 (day 7 after HI; the M7 group) without immunosuppressants. In total, 27 rats were assigned to either of the three groups (the vehicle, M3 and M7 groups) consisted of 9 HI rats, respectively.

#### Survival rate and body weight

All rats in the vehicle, M3, and M7 groups survived until the 10-week observation period. Repeated general observations indicated no adverse events.

**Figure 3:**
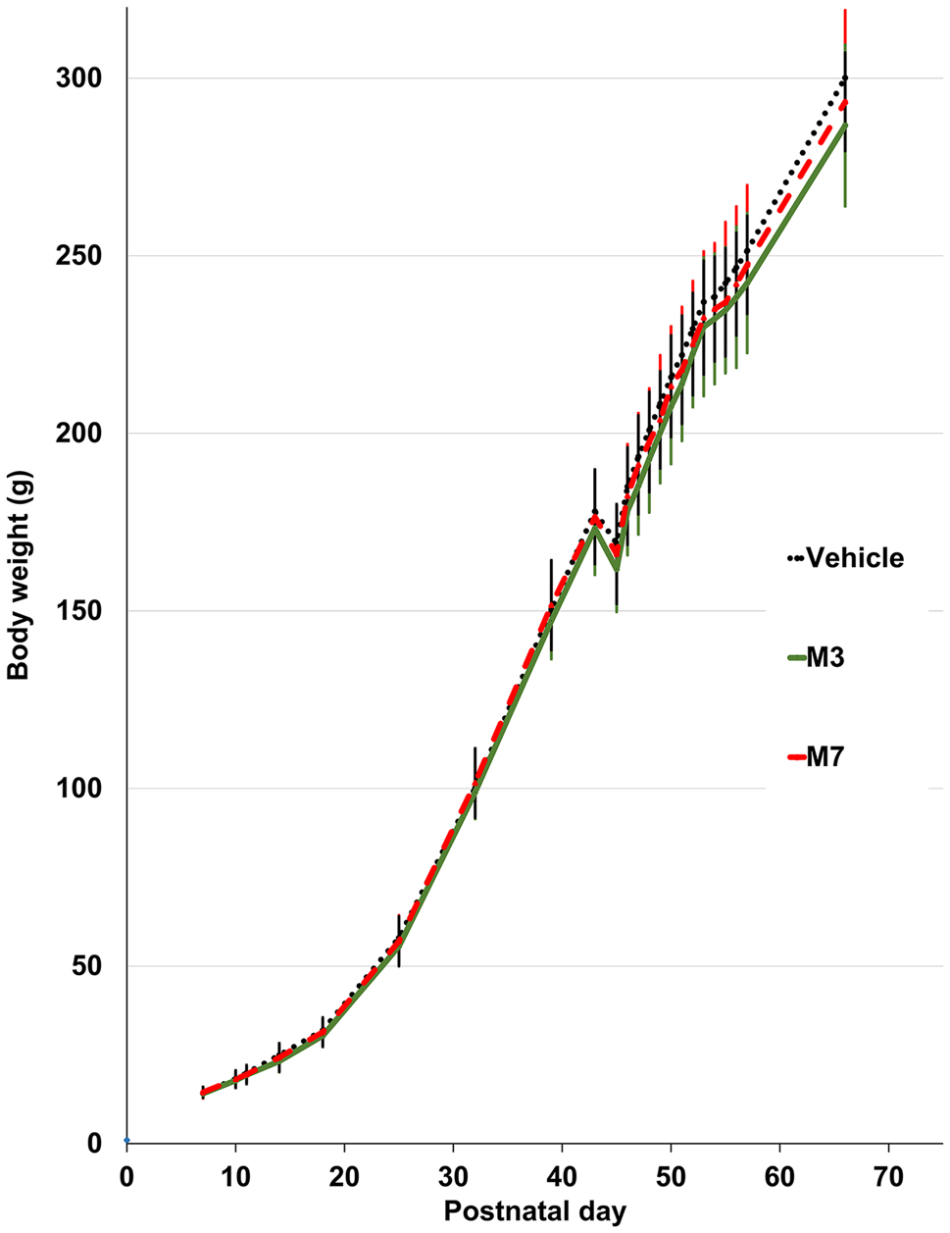
Body weight gain after birth throughout the observation period (*n* = 9 for the vehicle group, *n* = 9 for the M3 group, and *n* = 9 for the M7 group). Black dotted line, vehicle; green solid line, M3; red dashed line, M7. Data represent mean ± standard deviation.

Body weight gain is shown in Fig. 3. Temporary body weight loss was observed in all three groups from P43 to P45, probably because of animal transportation as described in Experimental protocol, Method section. Statistical analysis indicated no significant difference among the three groups.

#### Cylinder test

**Figure 4:**
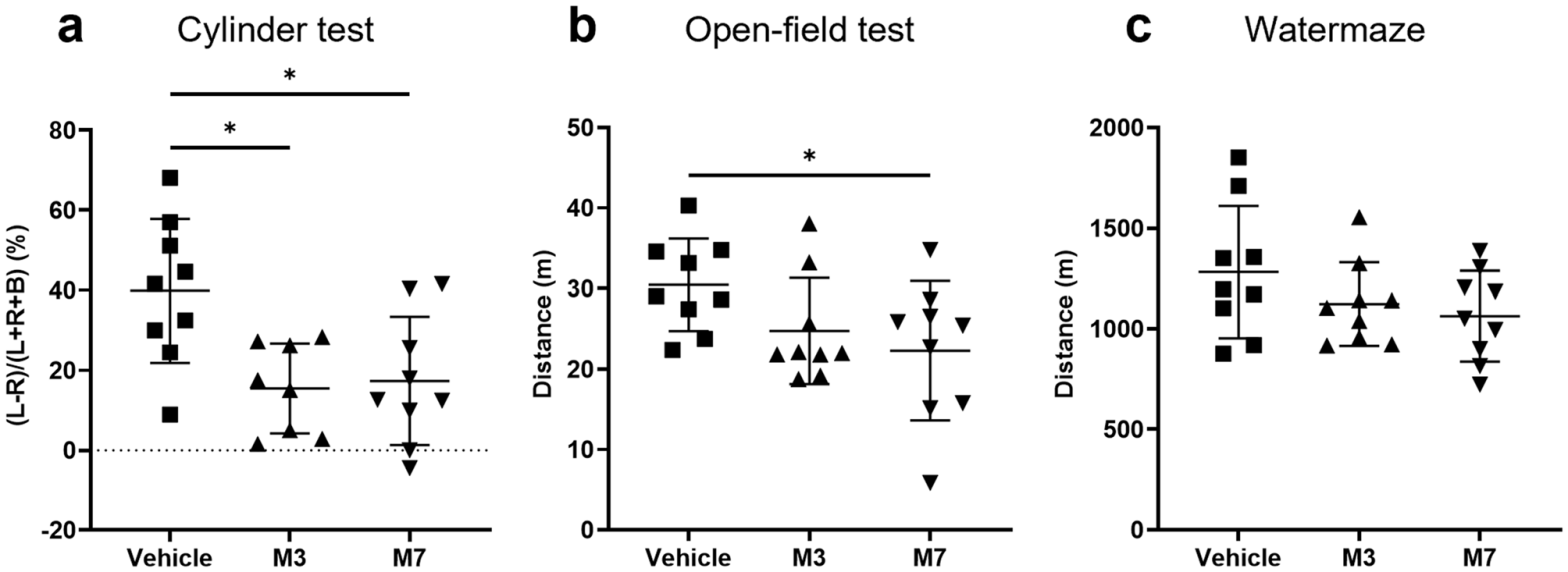
(**a**) In the cylinder test, the average of the preference for the left (ipsilateral) forepaw was calculated on three consecutive days (postnatal day 36 (P36) to P38 (*n* = 9 for the vehicle group, *n* = 8 for the M3 group, and *n* = 9 for the M7 group). Rats in the M3 and M7 groups showed a significantly lower preference for the ipsilateral (left) forepaw than rats in the vehicle group. (**b**) In the open-field test, the distance traveled was evaluated on P42 (*n* = 9 for vehicle, *n* = 9 for M3, and *n* = 9 for M7). The distance traveled was significantly shorter in the M7 group than in the vehicle group. (**c**) In the water maze test, the average of the distance traveled on five consecutive days (P53–P57) was calculated (*n* = 9 for vehicle, *n* = 9 for M3, and *n* = 9 for M7). Data represent mean ± standard deviation. *p < 0.05 and **p < 0.01.

The cylinder test was conducted to assess forelimb use preference from P36 to P38. The average values of the forelimb use preference in the M3 and M7 groups were significantly smaller than that in the vehicle group (Fig. 4a; *p* < 0.01, for both M3 vs. vehicle and M7 vs. vehicle). There was no significant difference between the M3 and M7 groups.

#### Open-field test

The open-field test was performed to assess hyperactivity on P42. The distance traveled in the M7 group was significantly shorter than that in the vehicle group (Fig. 4b; *p* < 0.05). There was no statistical significance between the vehicle and M3 groups (Fig. 4b).

#### Water maze test

The water maze test was conducted to assess spatial learning and memory from P53 to P57. There was no significant difference between the vehicle and M3 or M7 groups for the distance traveled (Fig. 4c).

#### Brain weight

There was no significant difference in brain weight between the vehicle group and the M3 or M7 groups (Supplemental Fig. 3).

#### Histopathological examination of the brain

**Figure 5:**
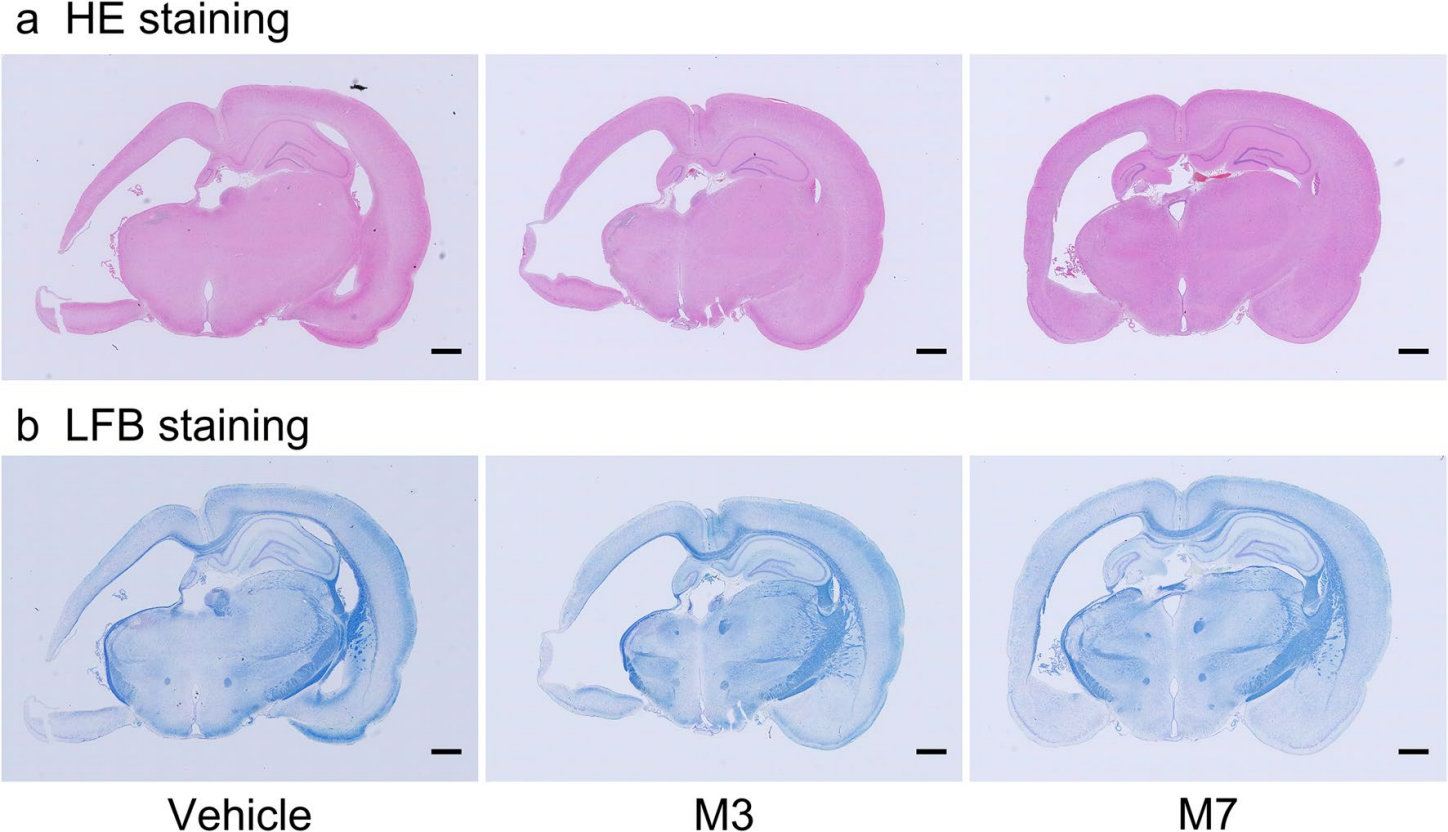
Representative images showing brain sections stained with hematoxylin–eosin (HE; **a**) and Luxol fast blue (LFB; **b**). (**a**) Enlargement of ventricles; vehicle group (grade 3); M3 group (grade 4); M7 group (grade 3). Bar = 1 mm. (**b**) Atrophy of nerve fascicles in the corpus callosum, external capsule, alveus of hippocampus, and fimbria of hippocampus; vehicle (grade 3); M3 (grade 3); M7 (grade 3). Bar = 1 mm.

**Figure 6:**
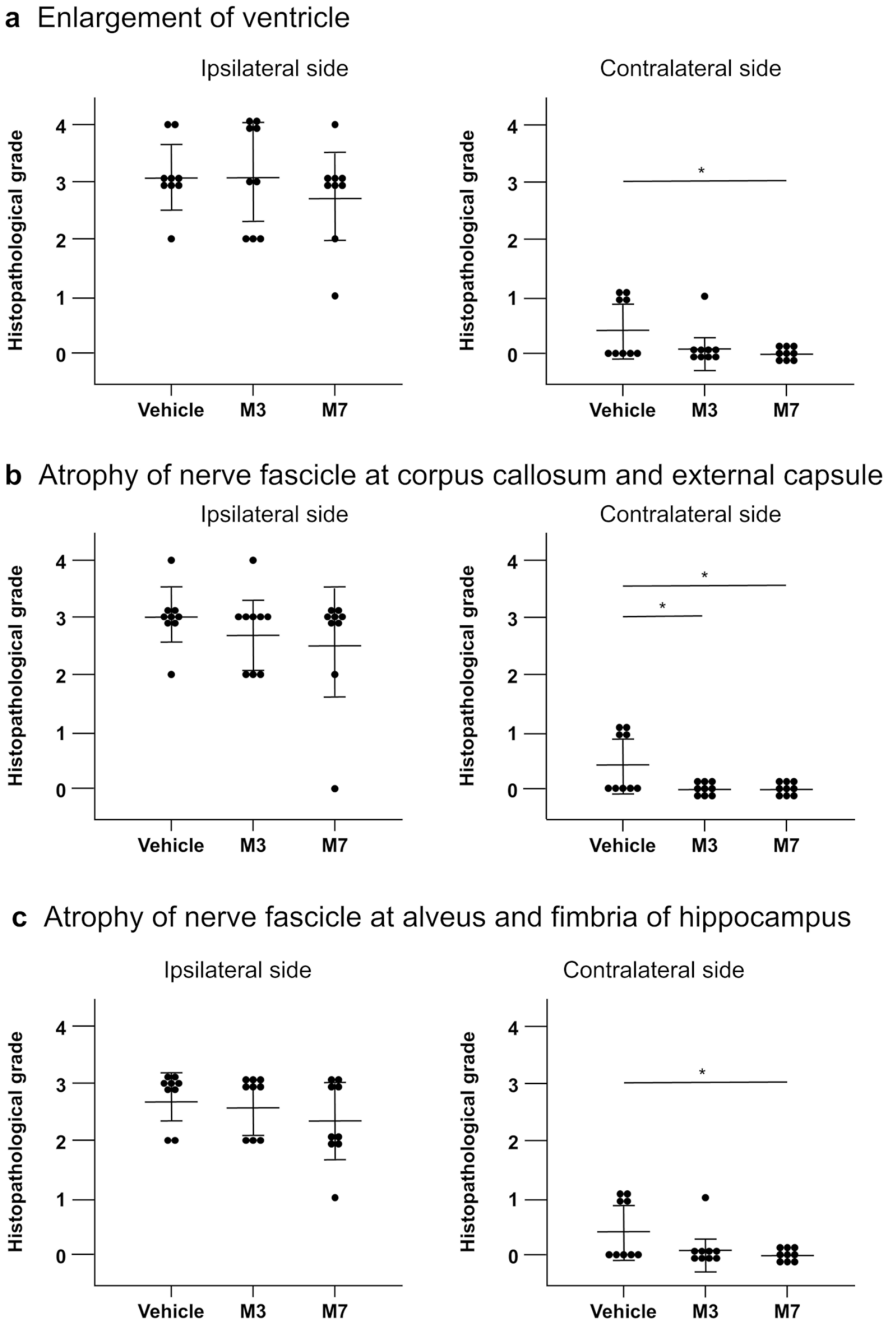
Histopathological grade of enlargement of ventricles on ipsilateral (left) and contralateral sides (**a**), atrophy of nerve fascicles in the corpus callosum and external capsule on ipsilateral and contralateral sides (**b**), and atrophy of nerve fascicles in the alveus of hippocampus and fimbria of hippocampus on ipsilateral and contralateral sides (**c**); *n* = 9 for the vehicle group, *n* = 9 for the M3 group, and *n* = 9 for the M7 group. Data represent mean ± standard deviation. *p < 0.05 and **p < 0.01.

Enlargement of ventricles on both the ipsilateral (left) and contralateral sides was assessed using HE staining (Fig. 5a). Figure 5a shows representative images for the vehicle, M3, and M7 groups. There was no significant difference in the average histopathological grade between the vehicle group and the M3 or M7 group on the ipsilateral side (Fig. 6a). However, on the contralateral side, the average histopathological grade in the vehicle group was significantly higher than that in the M7 groups (Fig. 6a; *p* < 0.05, vehicle vs. M7).

LFB staining revealed atrophy of nerve fascicles in the corpus callosum, external capsule, alveus of hippocampus, and fimbria of hippocampus (Fig. 5b). Atrophy was observed in the vehicle, M3, and M7 groups (Fig. 6b and 6c). In the ipsilateral corpus callosum and external capsule, there was no significant difference in the average histopathological grade between the vehicle group and the M3 or M7 group (Fig. 6b). However, in the contralateral corpus callosum and external capsule, the average histopathological grade in the vehicle group was significantly higher than that in the M3 and M7 groups (Fig. 6b; *p* < 0.05 for vehicle vs. M3, and vehicle vs. M7).

In the ipsilateral alveus of hippocampus and fimbria of hippocampus, there was no significant difference in the average histopathological grade between the vehicle group and the M3 or M7 group (Fig. 6c). In the contralateral alveus of hippocampus and fimbria of hippocampus, the average histopathological grade in the vehicle group was significantly higher than that in the M7 groups (Fig. 6c; *p* < 0.01 for M7 vs. vehicle).

#### Histopathological examination of the lung

Histopathological analysis of the lung at 10 weeks showed no significant pathological changes such as ischemic changes, bleeding, inflammation, or tissue destruction in the vehicle, M3, and M7 groups, suggesting that nafimestrocel administration did not induce any embolism or other adverse effects (Supplemental Fig. 4).

#### Microglial evaluation

To assess microglial activation, microglia purchased from Cosmo Bio (COS-NMG-6-3C, Cosmo Bio Co.) were cocultured with nafimestrocel for 24 h, and then lipopolysaccharide (LPS) was added. Quantitative polymerase chain reaction (qPCR) was conducted at 3 or 24 h.

**Figure 7:**
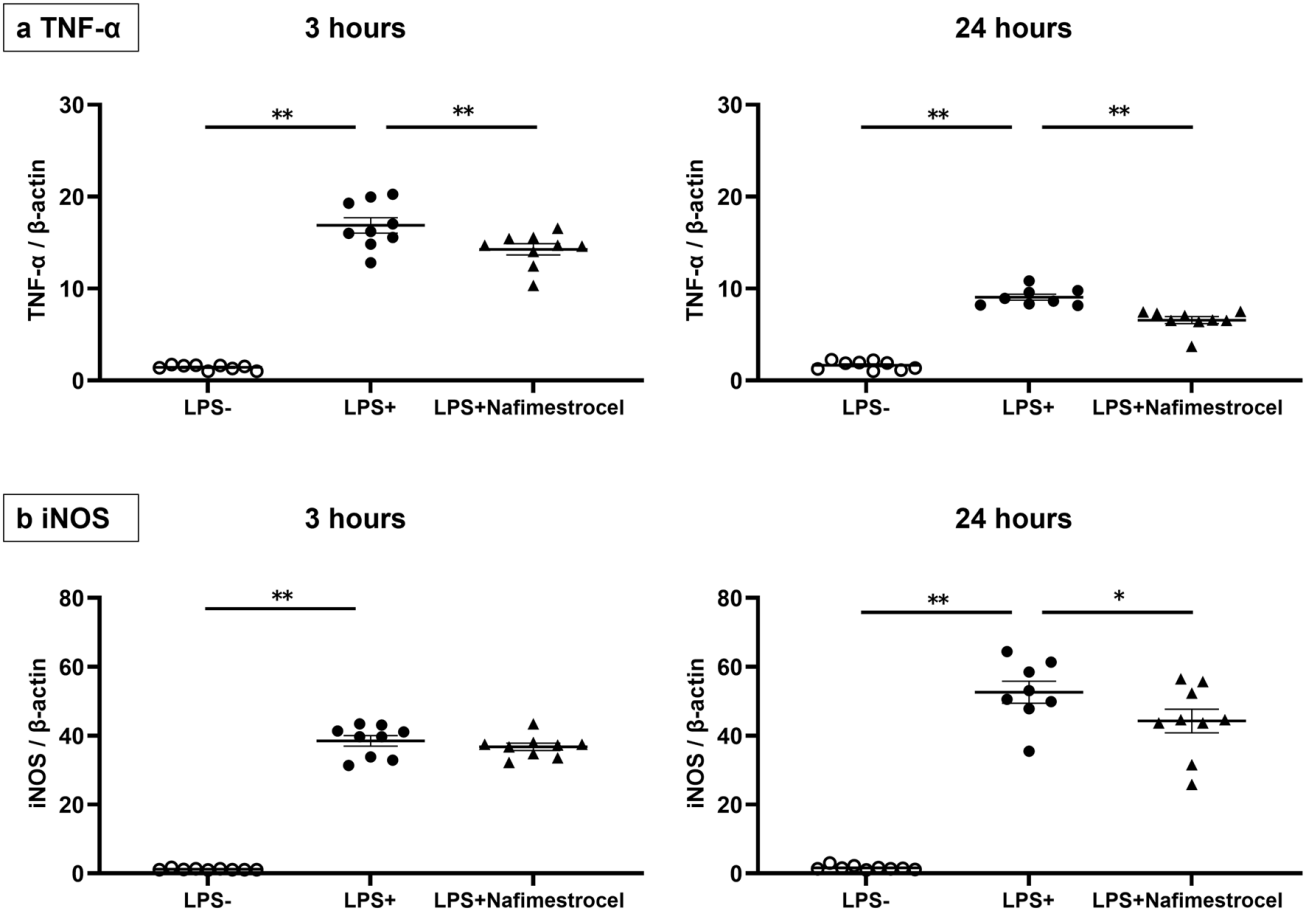
In vitro experiment using a coculture of microglia and nafimestrocel at 3 h (left) and 24 h after LPS administration. The production of TNF-α (**a**) and iNOS (**b**) was evaluated at 3 and 24 h after LPS administration. (**a**) The production of TNF-α was significantly lower in microglia cocultured with nafimestrocel at both 3 and 24 h after LPS administration. (**b**) The production of iNOS significantly decreased in microglia cocultured with nafimestrocel at 24 h after LPS administration. White circles (LPS −) correspond to microglia alone without LPS, black circles (LPS +) represent microglia alone with LPS, and black triangles (LPS + nafimestrocel) indicate microglia cocultured with nafimestrocel and LPS (*n* = 9 for LPS − , 9 for LPS + , 9 for LPS + nafimestrocel at 3 h, and *n* = 9 for LPS − , 8 for LPS + , 9 for LPS + nafimestrocel at 24 h). Data represent mean ± standard deviation. *p < 0.05 and **p < 0.01.

The production of TNF-α was significantly increased at 3 h after LPS administration (Fig. 7a; *p* < 0.01, LPS + vs. LPS −) and was suppressed by coculture with nafimestrocel (Fig. 7a; *p* < 0.01, LPS + vs. LPS + nafimestrocel), and this effect was maintained at 24 h (Fig. 7a; *p* < 0.01, LPS + vs. LPS + nafimestrocel) after LPS administration. Similarly, the production of iNOS was significantly increased both at 3 h and 24 h after LPS addition (Fig. 7b;* p* < 0.01, LPS + vs. LPS − at 3 h and 24 h). The presence of nafimestrocel did not change the production of iNOS at 3 h, but significantly decreased the iNOS level at 24 h, after LPS administration (Fig. 7b; *p* = 0.040, LPS + vs. LPS + nafimestrocel).

## Discussion

In the present study, systemically administered nafimestrocel, cells suspended from a clinical-grade human Muse cell-based product (CL2020), homed to the injured brain tissue after HI and was shown to remain in the brain at 168 h after administration in a rat model. Nafimestrocel-treated rats showed statistically significant improvement compared with the vehicle group in the cylinder test (both the M3 and M7 groups) and in the open-field test (the M7 group). In the water maze test, neither of M3 or M7 group attained significant improvement though the transportation and temporary body weight loss might have affected the result adversely. Moreover, the nafimestrocel-treated rats did not exhibit adverse effects such as death, weight loss, or histopathological changes in the lung throughout the observation period for up to 10 weeks. Histopathological examination of the brain suggested that nafimestrocel attenuated the enlargement of ventricles on the contralateral side in the M7 group. Atrophy of nerve fascicles on the contralateral side was alleviated in the corpus callosum, external capsule in the M3 and M7 groups, while atrophy of nerve fascicles on the contralateral alveus of hippocampus and fimbria of hippocampus was significantly improved in the M7 group. qPCR demonstrated that nafimestrocel suppressed the production of the inflammatory mediators TNF-α at 3 and 24 h and iNOS at 24 h in vitro. These results indicate that nafimestrocel exerted its therapeutic effects in some aspects without serious adverse events.

In the present study, autoradiography revealed homing and survival of nafimestrocel in HI brains, and also suggested that homing was not only restricted to the ipsilateral side but also extended to the contralateral side. Muse cells are known to selectively home to damaged tissue using the S1P–S1PR 2 axis^19^. S1P is produced by various tissues upon damage, and its pathological roles in neurological disorders have also been reported^23,36^. The Rice–Vannucci model used in the present study with minor modifications is known to involve damage on both the ipsilateral and contralateral sides^37^. While only the left common carotid artery was disconnected in this model, brain injury was induced in the both hemispheres due to the systemic hypoxia by placing animals under hypoxia for 60 min after cutting the left common carotid artery. For this, nafimestrocel might have homed to both cerebral hemispheres in HI rats.

Tissue damage was more serious in the ipsilateral hemisphere rather than in the contralateral hemisphere. In histological data, the tissue loss in the ipsilateral hemisphere was more sever and higher than that in the contralateral hemisphere (Fig. 5). Probably because of this, the M3 and M7 groups could not show better histopathologial grade compared to the vehicle group in the ipsilateral side while better improvement was shown in the contralateral side (Fig. 5). Nevertheless, the fact that nafimestrocel homed to both sides of the brain and exhibited therapeutic effects in some but not all evaluations might be advantageous, considering the problem of low engraftment with administration of various stem or progenitor cells in central nervous system disease models^38^.

One of the key findings of the present study is that nafimestrocel, a xenogeneic clinical-grade human Muse cell-based product (CL2020) cells, worked in a rat model of HIE without using any immunosuppressive agent for up to 10 weeks. This finding is consistent with our previous findings based on FACS-isolated research-grade human Muse cells in a rat model of HIE^32^. Human Muse cells express HLA-G and interferon gamma-induced indoleamine-2,3 dioxygenase, known to exert immunomodulation^19,20^. The system enables Muse cells to survive as functional cells for over 6 months after administration without immunosuppressants, as demonstrated in a rabbit model of acute myocardial infarction^19^. These findings suggest that immunosuppression is not required for the treatment with nafimestrocel, an allogeneic clinical-grade, human Muse-cell product (CL2020) cells. In two human clinical trials for the treatment of acute myocardial infarction and dystrophic epidermolysis bullosa, intravenous administration of CL2020 was successfully performed without immunosuppression^30,31^. It is advantageous for infants with HIE, who are clinically immunocompromised and need to undergo hypothermia treatment, a standard therapy for neonatal HIE known to weaken immune competency^39^.

Moreover, the M7 group who received nafimestrocel administration at seven days after HI insult (P14) showed therapeutic effects in the cylinder and open-field tests rather than the M3 group who were treated at 3 days after HI insult. Survival and neuronal differentiation of administered cells strongly depend on the timing of administration. Sato et al^40^ showed that early transplantation of neural stem cells at 24 h after the irradiation brain injury resulted in the reduction in surviving cells and promotion of astroglial differentiation, whereas favorable survival and neural differentiation were initiated by later transplantation, namely, one or six weeks after insult^32^. In the present study, the histopathological evaluation of the contralateral side in HI rats also revealed that nafimestrocel was more effective in the M7 group rather than M3 group. This finding offers an advantage in clinical application, since several days are required to stabilize newborns with HIE and complete hypothermia therapy. Besides, already existing medical drugs, such as magnesium sulfate and edaravone, have not achieved a significant neuroprotective outcome in HIE, in part because most of their effects are limited only to the acute phase^41–43^. Abe et al.^28^ reported that intravenous administration of Muse cells at 9 or 30 days after infarction was effective in a lacunar stroke model in adult mice, suggesting that Muse cells elicit their pleiotropic effects once they home to the damaged brain tissue even at the subacute phase^28^. Regarding brain development in rodents and humans, a rat brain at P14 is estimated to correspond to the brain of approximately 3-month-old infants in humans^44,45^. Collectively, the findings suggest that nafimestrocel may deliver curative effects at several weeks after the onset of neonatal HIE.

We evaluated the safety of nafimestrocel based on mortality, body weight, and the pathological assessment of the lung. Some reports suggest that the intravenous administration of cells increase risks of mortality and pulmonary embolism^13,46^. Growth is a factor as important as mortality from the viewpoint of side effects in newborns and infants. The present findings in a rat model do not suggest that nafimestrocel induces increased mortality, lung embolism, or growth retardation. Moreover, our observations revealed no apparent adverse events for up to 10 weeks. The safety profile of Muse cells has been reported in immunodeficient animals^16,20^. The present study suggested the safety of nafimestrocel using vulnerable, newborn rats, which would contribute to its future application in HIE and various other neonatal diseases.

The present study has certain limitations. First, only male P7 rats were used as HI model animal. In rodents, it is recently proposed that the P10 rat brain is more equivalent of a term human brain^47^. Meanwhile, this research was conducted as a non-clinical study to proceed to a clinical trial, and P7 rats are more widely used to test therapeutic interventions for HIE and neurological impairments^48,49^. It is also noted that many studies on stem cell therapy for neonatal brain injury have utilized P7 rats as test subject^50^. Further investigation is needed to clarify the treatment effects both for HI rats close to term human brain and female rats. Second, we did not evaluate its therapeutic effect in combination with hypothermia. Hypothermia has been clinically used to prevent reperfusion injury for 72 h, and the effect of the combination or interaction should be examined^51,52^. However, our study is potentially important in that nafimestrocel administration showed therapeutic effects, particularly at seven days after HI insult. Third, the precise mechanism of the therapeutic effects of nafimestrocel was not fully clarified. Muse cells possess various advantageous properties including specific homing to damage site after intravenous injection, spontaneous differentiation into tissue-constituent cells in vivo, delivering trophic, anti-inflammatory, anti-apoptotic and immunomodulatory effects, and long-term engraftment^15,18,19^. These properties may comprehensively lead to the curative effects in HIE, whereas the molecular biological approach in the present study was limited. We previously showed that Muse cells inhibited excitotoxic brain glutamatergic metabolites and suppressed microglial activation^32^. In the present study, we also demonstrated that nafimestrocel suppressed the production of TNF-α and iNOS induced by microglial activation, which is consistent with our previous findings based on research-grade Muse cells^32^. Moreover, further investigation is needed to identify the optimal timing for administration. In the present study, two time points, three and seven days after onset, for administration were evaluated. Other time points, for example, later than seven days, and/or comparison between single dose vs multiple doses should be examined in the future. These results provide a basis for assessing the safety and tolerability of nafimestrocel in newborns with HIE, and may have allowed us to consequently start clinical trials of nafimestrocel for patients with HIE (trial registration numbers: NCT04261335, and jRCT2043190112)^53^.

## Conclusion

In conclusion, nafimestrocel systemically administered at both three and seven days after HI without immunosuppression homed to HI brain tissue and demonstrated potential curative effects on behavioral and histopathological damage without any adverse events. Our findings suggest the feasibility of intravenous administration of nafimestrocel in the treatment of human HIE.

## Methods

A detail description of Materials and Methods is available in the online Supplement.

### Ethics approval

All experiments were approved by the Animal Care and Use Committee of Nagoya University School of Medicine (Nagoya, Japan; permit No.: 30079), Fukuoka University (Fukuoka, Japan; permit No.: 1712121), Sekisui Medical Co. Ltd. (Tokyo, Japan; No.: 2018–070), or BoZo Research Center Inc. (Tokyo, Japan; No.: T180090) and were conducted in accordance with the Regulations on Animal Experiments in Nagoya University. The present study is reported in compliance with the ARRIVE guidelines (Animal Research: Reporting in Vivo Experiments).

### Animals

A total of 33 male Wistar/ST rat pups were used in this study. They were obtained from Japan SLC Inc. (Shizuoka, Japan) and housed in a temperature-controlled room (23 °C) on a 12 h light/dark cycle with food and water ad libitum.

Three animals were allocated to sham used for radioisotope experiment, and 30 animals were exposed to HI. Of these, 27 were used for behavioral and histological evaluation, and 3 for radioisotope experiment.

The minimum sample size for the behavioral and histological evaluations was calculated based on the preliminary experiments to achieve an 80% power of testing with an error rate of 1.67%, assuming a 17% difference and 10% standard deviation in the cylinder test as a primary endpoint.

The total sample size was calculated as n = 24 in this case. Additionally, in our previous studies, a few rats would occasionally die, particularly during the process of creating the model, though all rats in this study survived finally. Therefore, the number of rats was set to 9 per each group for behavioral and histological evaluations.

### Hypoxic–ischemic insult

Hypoxic–ischemic brain injury was induced on P7 using the modified method described by Rice et al^32,54^. In brief, the left common carotid artery was doubly ligated and was incised at the site between the ligatures under anesthesia with isoflurane. After 1 h rest, the pups were placed in a hypoxic environment (8% O_2_ and 92% N_2_ at 37 °C for 60 min). The sham group for radioisotope experiment underwent only anesthesia and identification of the left carotid artery without ligation or hypoxia.

### Assessment of the injury with MRI

Diffusion-weighted MRI was performed 3–5 h after HI insult (P7) for the assignment of rats to the radioisotope experiment. Sham rats for radioisotope experiment did not undergo MRI. T2-weighted MRI was performed three days after HI insult (P10) for the assignment of rats to evaluation of safety, behavior, and histopathology.

The severity of brain injury was categorized into three grades using the method described by Mikrogeorgiou et al.^55^. (Supplemental Fig. 1a and 1b): mild (no or little hyperintensity in the parietal cortex), moderate (unilateral hyperintensity occupying the cortex and hippocampus), or severe (unilateral hyperintensity occupying the cortex and hippocampus and extending to the striatum and basal ganglia). Rats with mild HI injury were deemed unsuitable because the damage was too low, whereas those with severe HI injury were considered unsuitable because the damage was too high and could result in diffuse necrosis and ipsilateral brain collapse (Supplemental Fig. 2). Rats with moderate HI injury showed close equivalence to human HI. Therefore, moderately injured rats were adapted for subsequent experiments (Supplemental Fig. 2).

### In vivo* dynamics of intravenously administered nafimestrocel by using radioisotopes*

#### ^**51**^Cr labeling of nafimestrocel

Nafimestrocel, produced from human MSCs by exposing the cells to a combination of stresses, was supplied by Life Science Institute, Inc. (Tokyo, Japan)^28,30^. The cell concentration was adjusted to 1 × 10^7^ cells/mL with Hank’s balanced salt solution (HBSS) for administration. Tracer (chromium-51 radionuclide, 185 MBq/mL; PerkinElmer Inc., Waltham, MA) was used for radiolabeling.

#### ^**51**^Cr-labeled nafimestrocel administration

On P10, the pups received intravenous administration of ^51^Cr-labeled nafimestrocel (1 × 10^6^ cells/body) or HBSS (0.1 mL/body) via the right external jugular vein under the anesthesia with isoflurane.

### Preparation of brain samples

We established three time points to examine the distribution of radioactivity in rat brain: 24, 72, and 168 h after intravenous injection of ^51^Cr-labeled nafimestrocel. The frozen coronal brain Sects. (30 µm thickness) were prepared in two planes: one plane contained the striatum and the other contained the hippocampus and optic thalamus. The radioactivity from each section was acquired to obtain radioluminograms.

Regions of interest (ROIs) were established along the periphery of cerebral parenchyma. Radioactivity in each ROI was calculated as photostimulated luminescence (PSL) per unit area (PSL/mm^2^). In HI rats, the remaining parenchyma in the ipsilateral and contralateral hemispheres was evaluated. In sham rats, the whole brain was considered as the ROI to detect the smallest amount of radioactivity, and total radioactivity was calculated.

### Assignment of groups

We established two time points for cell administration—three and seven days after HI insult (P10 and P14, respectively)—to verify its therapeutic effect. The cell number of injected nafimestrocel was 1 × 10^6^ cells/body, suspended in 0.1 mL HBSS. HI rats were allocated to three groups: the M3 group (*n* = 9) received nafimestrocel administration on P10 and then followed by the HBSS injection (0.1 mL/body) on P14; the M7 group (*n* = 9) received an HBSS injection on P10 and then followed by nafimestrocel administration on P14; and the vehicle group (*n* = 9) received HBSS injections on P10 and P14.

### Preparation of nafimestrocel for administration

Nafimestrocel for administration was prepared in the same manner as described above. Nafimestrocel was suspended in HBSS at a concentration of 1 × 10^7^ cells/mL.

### Nafimestrocel administration

The pups received an intravenous injection of nafimestrocel (1 × 10^6^ cells/0.1 mL) or HBSS (0.1 mL/body) on P10 and P14 according to the group assignment. No immunosuppression was performed throughout this study.

### Behavioral tests

Grouping for all behavioral tests and evaluations was done blindly.

### Cylinder test

The cylinder test was performed to assess forelimb use preference from P36 to P38 consecutively using the modified method of Schallert et al.^56^. The forelimb use preference was calculated as follows: (nonimpaired − impaired)/(nonimpaired + impaired + both) × 100. The average value in each rat was used for statistical analysis.

### Open-field test

The open-field test^57^ was conducted on P42 to assess hyperactivity. Each rat was placed in the center of an open-field chamber, and the distance traveled was recorded for 5 min using the ANY-maze Video Tracking System (Stoelting Co., Wood Dale, IL).

### Water maze test

The water maze test was conducted from P53 to P57 consecutively with Morris water maze pool (Neuroscience®) and WaterMaze™ software (Actimetrics, Wilmette, IL), which was modified in accordance with Morris et al.^58,59^. Trial was performed three times per day. Distance traveled was recorded with software. The average from all five days was analyzed.

### Pathological examination

Brains and lungs were collected at 10 weeks. Brains were weighed before tissue fixation. Paraffin-embedded brain sections including the cerebral cortex, hippocampus, thalamus, and basal ganglia were selected by referring to the Paxinos and Watson brain atlas (plate levels 92 and 93)^60^ and were evaluated. The sections were stained with hematoxylin–eosin (HE) and Luxol fast blue (LFB). Lung sections were randomly selected from six rats in each group and were stained with HE.

### Injury evaluation

A semiquantitative neuropathological scoring system using the modified methods of previous reports^61,62^ (Supplemental Table) was adopted, and each hemisphere was evaluated. The mean grade in each group was used for analysis.

### Microglial activation assessments

Microglial activation assessment was conducted based on the method reported by Suzuki et al. with minor modifications^32^. In brief, microglia (6–3 Microglia Cell Clone, COS-NMG-6-3C, Cosmo Bio Co., Ltd., Tokyo, Japan) were plated on 24-well plates at a density of 1.1 × 10^4^ cells/cm^2^ (2.0 × 10^4^ cells/well) using a medium (COS-NMGM, Cosmo Bio Co) containing 33 ng/mL recombinant mouse GM-CSF (415-ML-010/CF, R&D systems, Minneapolis, MN).

Nafimestrocel was seeded at a density of 1.8 × 10^4^ cells/cm^2^ (6 × 10^3^ cells/well) on transwell inserts (Boyden chamber: FALCON Cell Culture Insert, Corning Life Sciences, Corning, NY) and cultured in minimum essential medium Eagle, alpha modification (Thermo Fisher Scientific, Waltham, MA), with 10% fetal bovine serum (Thermo Fisher Scientific, Waltham, MA) and 1 ng/mL basic fibroblast growth factor (Miltenyi Biotec, Bergisch Gladbach, Germany) for 24 h. Subsequently, the inserts were transferred to the 24-well plates in which microglia were cultured for 24 h before LPS administration. The inserts with nafimestrocel were removed six days after starting the culture, and LPS (serotype O55:B5, Sigma-Aldrich, St. Louis, MO) or phosphate-buffered saline was added to microglial cultures at a concentration of 100 ng/mL. Total RNA was extracted from the microglial cultures at 3 and 24 h after adding LPS, considering microglial survival capability, and reverse transcription was performed using 200 ng of total RNA. Then, qPCR was performed by LightCycler 96 System (Roche Diagnostics, Indianapolis, IN) and KOD SYBR qPCR Mix (QKD-201, Toyobo Co., Ltd, Osaka, Japan) and following primers were used: tumor necrosis factor (TNF)-α sense, 5′-GTAGCCCACGTCGTAGCAAAC-3ʹ; antisense, 5ʹ-CTGGCACCACTAGTTGGTTGTC-3ʹ; iNOS sense, 5ʹ-CATGCTACTGGAGGTGGGTG-3ʹ; antisense, 5ʹ-CATTGATCTCCGTGACAGCC-3ʹ; β-actin sense, 5′-CGTGGGCCGCCCTAGGCACCA-3; and antisense, 5′-ACACGCAGCTCATTGTA-3^63^.

### Statistical analysis

Statistical analysis was performed by using SPSS software version 26 (SPSS Inc., Chicago, IL) and GraphPad Prism software version 9 (GraphPad Software, San Diego, CA). One-way analysis of variance, followed by Holm–Šídák’s multiple comparisons test, was used to assess body weight gain, behavioral test results, and microglial activation. Dunn’s test was used to analyze the findings of pathological examination. The value of M3 or M7 was compared with that of vehicle respectively. The Kaplan–Meier method and log-rank test with Bonferroni correction were employed to analyze the survival rate of rats. A *p*-value of < 0.05 was considered statistically significant. All values correspond to mean ± standard deviation.

### Supplementary Information

Supplementary Legends.

Supplementary Figure 1.

Supplementary Figure 2.

Supplementary Figure 3.

Supplementary Figure 4.

Supplementary Information 6.

Supplementary Table 1.

## Data Availability

The datasets and materials generated during the current study are available from the corresponding author upon reasonable request.
