# Supplementary material for: Systemic administration of clinical-grade multilineage-differentiating stress-enduring cells ameliorates hypoxic–ischemic brain injury in neonatal rats
Source: Sci Rep. 2023 Sep 11;13:14958. doi: 10.1038/s41598-023-41026-3 (PMC10495445; doi:10.1038/s41598-023-41026-3)
Supplement: Supplementary file 1 — Supplementary Legends. [file 41598_2023_41026_MOESM1_ESM.docx]

***Supplementary figure legends***

Supplementary figure 1. Examples of injury in 3 levels of the brain according to (a) diffusion-weighted MRI and (b) T2-weighted MRI. The severity of brain injury was categorized into 3 grades: mild (no or little hyperintensity in parietal cortex), moderate (unilateral hyperintensity occupying the cortex and hippocampus) or severe (unilateral hyperintensity occupying the cortex, hippocampus and extending to the striatum and basal ganglia). Hyperintensity of ipsilateral cortex (arrows) with additional hyperintensity in subcortical regions (hippocampus and basal ganglia, arrowheads).

Supplementary figure 2. Representative hematoxylin-eosin staining of brain (× 10). Bar = 1 mm.

Supplementary figure 3. Brain weight after 10-week observation (n=9 for Vehicle, 9 for M3 and 9 for M7). There was no significant difference between vehicle and the M3 or M7 group.

Supplementary figure 4. Representative hematoxylin-eosin staining of lung (× 200). (A)Vehicle; (B) M3; (C) M7.

There was no significant pathological change including embolism among all three groups. Bar = 50 μm.
