# Supplementary figures and images for: Systemic administration of clinical-grade multilineage-differentiating stress-enduring cells ameliorates hypoxic–ischemic brain injury in neonatal rats
Source: Sci Rep. 2023 Sep 11;13:14958. doi: 10.1038/s41598-023-41026-3 (PMC10495445; doi:10.1038/s41598-023-41026-3)

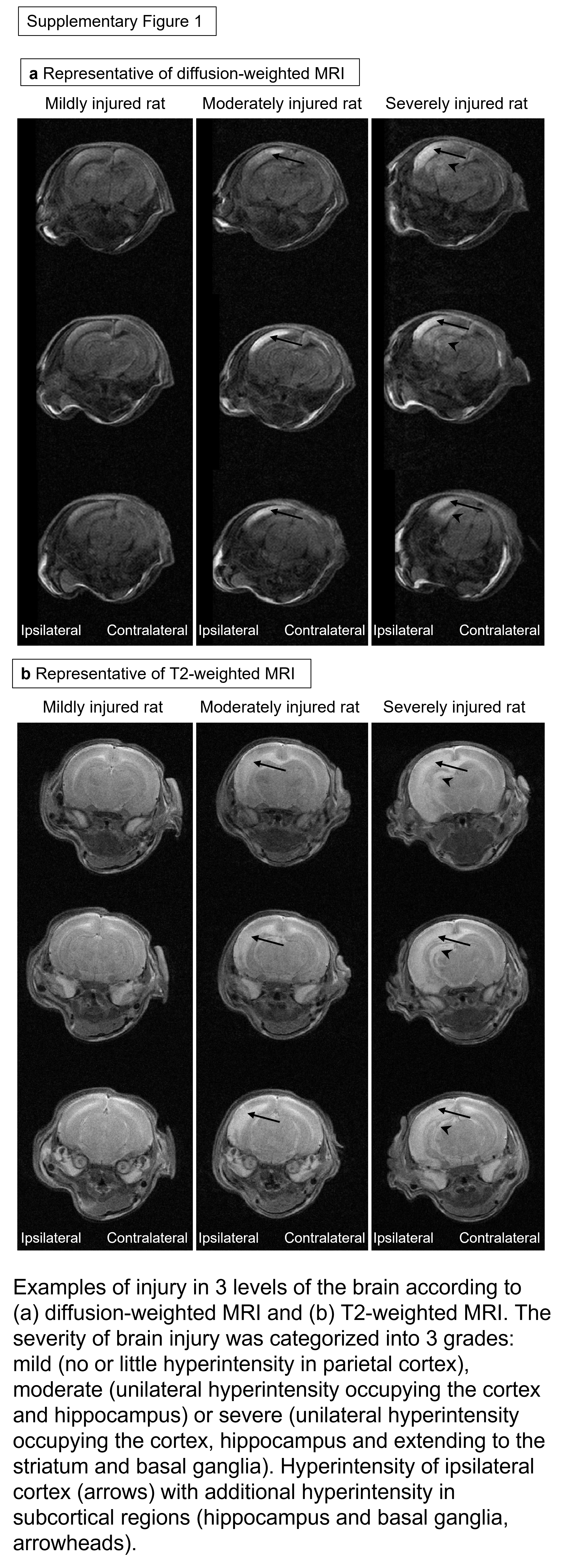

Supplement: Supplementary file 2 — Supplementary Figure 1. [file 41598_2023_41026_MOESM2_ESM.tif]

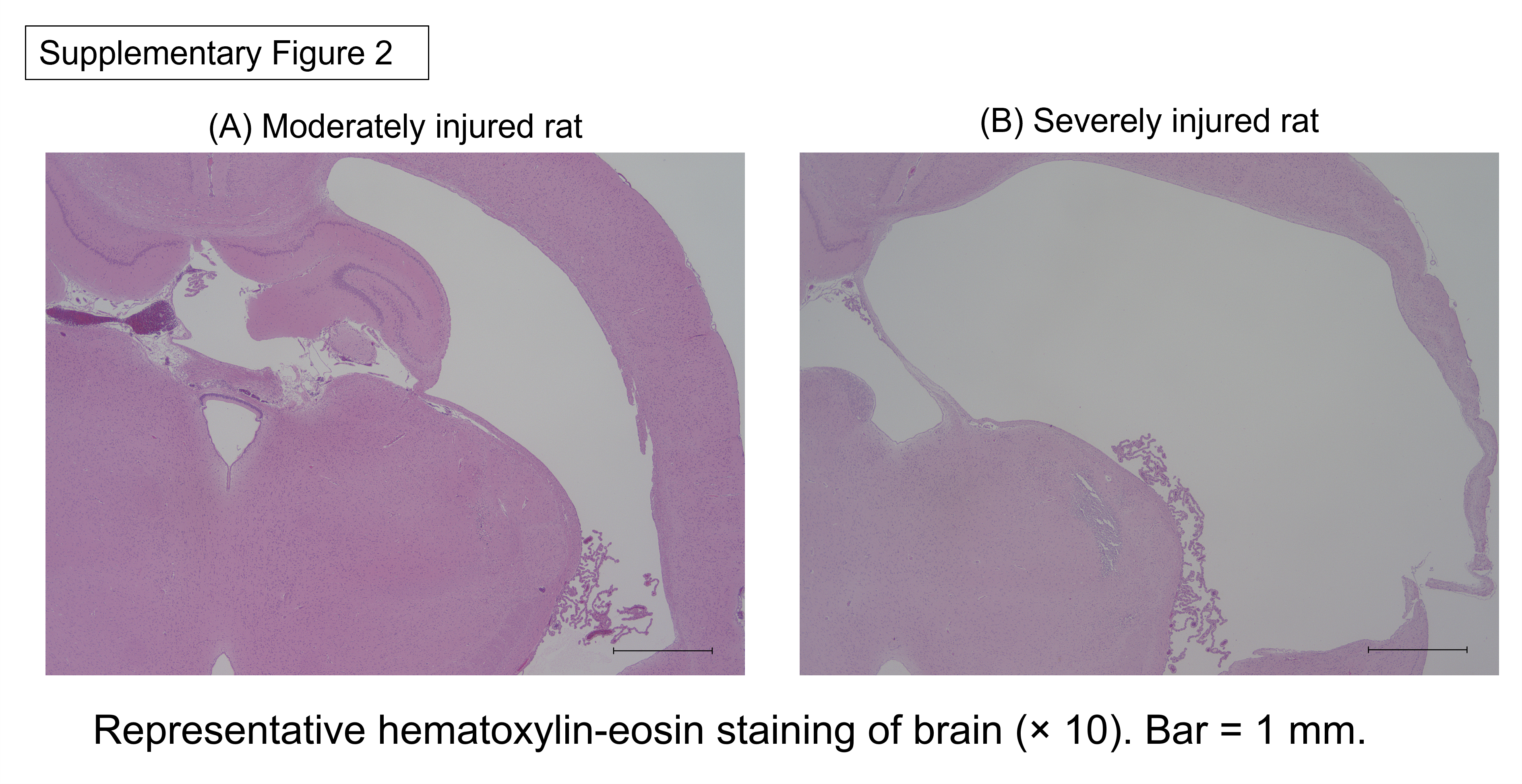

Supplement: Supplementary file 3 — Supplementary Figure 2. [file 41598_2023_41026_MOESM3_ESM.tif]

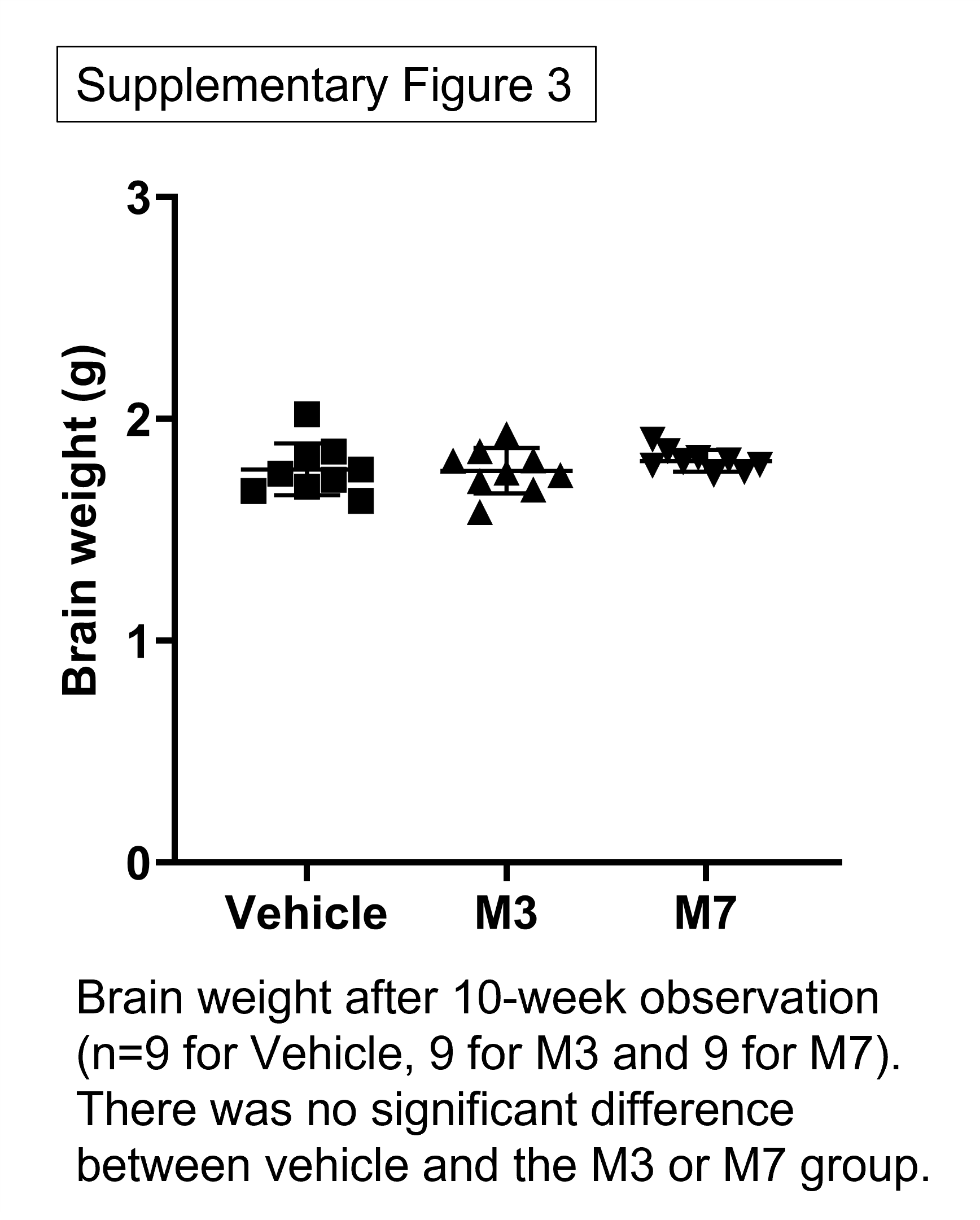

Supplement: Supplementary file 4 — Supplementary Figure 3. [file 41598_2023_41026_MOESM4_ESM.tif]

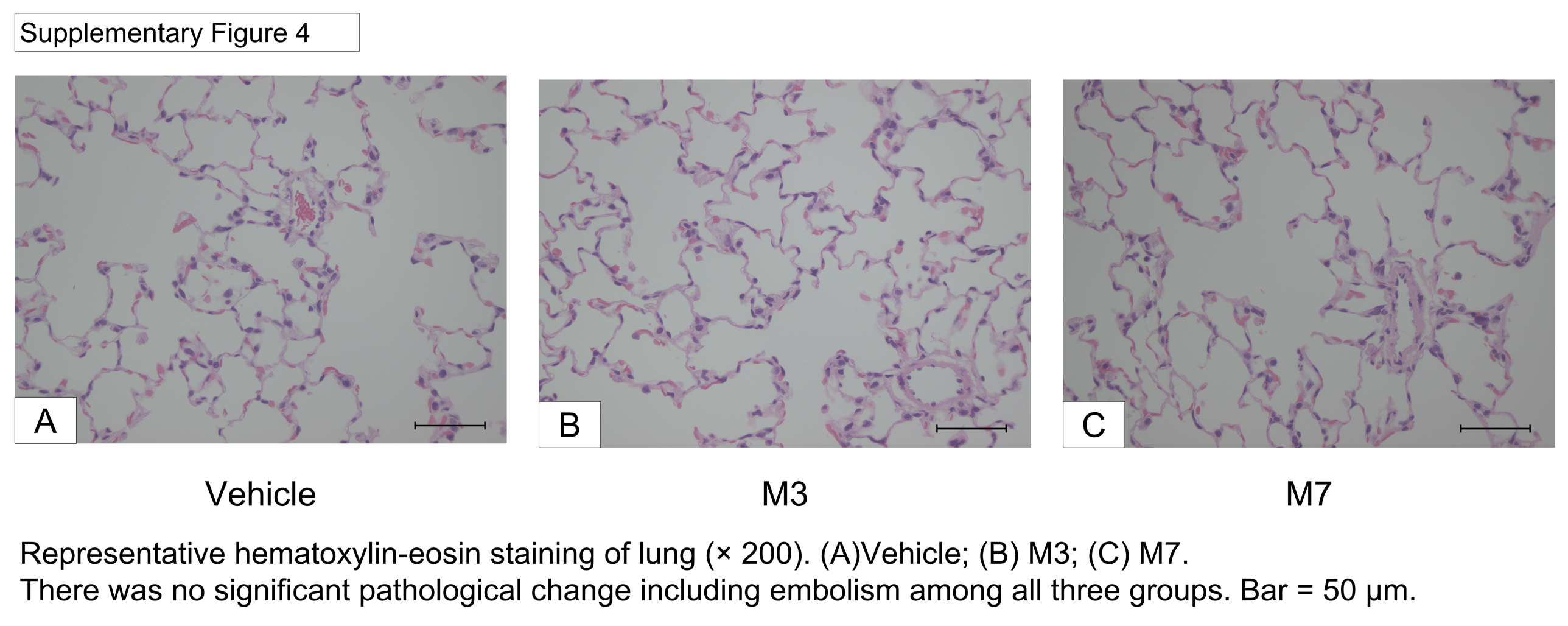

Supplement: Supplementary file 5 — Supplementary Figure 4. [file 41598_2023_41026_MOESM5_ESM.tif]
