# Supplementary material for: Systemic administration of clinical-grade multilineage-differentiating stress-enduring cells ameliorates hypoxic–ischemic brain injury in neonatal rats
Source: Sci Rep. 2023 Sep 11;13:14958. doi: 10.1038/s41598-023-41026-3 (PMC10495445; doi:10.1038/s41598-023-41026-3)
Supplement: Supplementary file 6 — Supplementary Information 6. [file 41598_2023_41026_MOESM6_ESM.docx]

**Supplementary methods**

***Experimental protocol***

Radioisotope experiment using chromium-51 radionuclide was performed using hypoxic–ischemic (HI) rat pups. HI insult was induced in male Wistar/ST rat pups on postnatal day 7 (P7). The severity of brain injury was evaluated by diffusion-weighted magnetic resonance imaging 3–5 h after HI insult. Based on clinical relevance (described later), only moderately injured rats were used in subsequent experiments. The criteria for moderate and severe injuries are described below. A single dose ^51^Cr-labeled nafimestrocel was administered intravenously on P10, and then the radioactivity in the brain was measured.

For safety assessment and behavioral experiments, HI brain injury was induced in the same manner. The severity of brain injury was evaluated via MRI on P10, and moderately injured rats were adapted as described below. Systemic administration of nafimestrocel was performed on either P10 (day 3 after HI) or P14 (day 7 after HI) without immunosuppressants. The cylinder and open-field tests were conducted from P36-38 and P42, respectively, at Nagoya University, after which the rats were transported to Fukuoka University, where the water maze test was performed from P53–P57. Subsequently, pathological examination of the brain and lung tissues was performed at ten weeks.

To assess microglial activation, microglia purchased from Cosmo Bio (COS-NMG-6-3C, Cosmo Bio Co.) were cocultured with nafimestrocel for 24 h, and then lipopolysaccharide (LPS) was added. Quantitative polymerase chain reaction (qPCR) was conducted at 3 or 24 h.

***Hypoxic–ischemic insult***

Hypoxic–ischemic brain injury was induced on P7 using the modified method reported by Rice et al ^33, 34^. In brief, each rat was anesthetized with isoflurane inhalation (2.5% both for induction and maintenance). The left common carotid artery was doubly ligated and incised between the ligatures. The pups were placed on a water bath (set to 37°C) before and after surgery and on a hot plate (set to 37°C) during surgical intervention to maintain their optimal body temperature. After 1 h rest with the dam, the pups were placed in a hypoxic environment (8% O_2_ and 92% N_2_ at 37°C for 60 min). Subsequently, the pups were returned to the dam in the animal room. The sham group for radioisotope experiment underwent only anesthesia and identification of the left carotid artery without ligation or hypoxia.

***Assessment of the injury with diffusion-weighted magnetic resonance imaging***

Diffusion-weighted MRI was performed 3–5 h after HI insult (P7) for assignment of groups to the radioisotope experiment. Rat brains were scanned on an MRS 3000/1500 Benchtop System (MR solutions, Guildford, UK) with a 3 T/17 cm cryogen-free superconducting magnet. Anesthesia was induced and maintained with 2.5% isoflurane during scanning. Animals were placed prone on a plastic holder and inserted into a quadrature birdcage imaging coil (20 mm in diameter). Eight coronal diffusion-weighted images were obtained to confirm each ipsilateral signal change suggesting HI injury with the following settings: repetition time (TR) = 1500 ms, echo time (TE) = 43 ms, field of view (FOV) = 20 mm^2^, slice thickness = 1.0 mm, slice separation gap = 2.0 mm, and average acquisition time = 10 min. Application software (Easyscan, MR Solutions, Guildford, UK) was used for image analysis.

The severity of brain injury was categorized into three grades on the basis of the method described by Mikrogeorgiou et al ^35^. (Supplemental Fig. 1a and 1b): mild (no or little hyperintensity in the parietal cortex), moderate (unilateral hyperintensity occupying the cortex and hippocampus), or severe (unilateral hyperintensity occupying the cortex and hippocampus and extending to the striatum and basal ganglia). Rats with mild HI injury were deemed unsuitable because the damage was too low, whereas those with severe HI injury were considered unsuitable because the damage was too high and could result in diffuse necrosis and ipsilateral brain collapse (Supplemental Fig. 2). Rats with moderate HI injury showed close equivalence to human HI. Therefore, moderately injured rats were adapted for subsequent experiments (Supplemental Fig. 2). Sham rats did not undergo MRI.

***In vivo dynamics of intravenously administered nafimestrocel by using radioisotopes***

Radiolabeling and tracking with chromium-51 radionuclide, modified from previous reports ^36-38^, was used to examine cell homing.

**^51^Cr labeling of nafimestrocel**: Nafimestrocel, produced from human mesenchymal stem cells by exposing the cells to a combination of stresses, was supplied by Life Science Institute, Inc. (Tokyo, Japan) ^29, 31^. The frozen bag including nafimestrocel was placed in a heating bucket (37°C), and the thawed cell suspension was placed into a centrifuging tube. HBSS (15 mL) was added, and the cell suspension was centrifuged at 700 × *g* for 5 min at room temperature. After removal of the supernatant, 5 mL HBSS was added, and the mixture was centrifuged again in the same manner. The supernatant was removed, and 100 µL tracer (chromium-51 radionuclide, 185 MBq/mL; PerkinElmer Inc., Waltham, MA) and 900 µL HBSS were added to the pelleted nafimestrocel. The cells were incubated for 1 h at 37°C. Centrifugation was performed twice under the conditions described above. The cells were resuspended in HBSS, and the number of cells and survival rate were confirmed by staining with 0.4% trypan blue. The cell concentration was adjusted to 1 × 10^7^ cells/mL for administration. Some of the ^51^Cr-labeled nafimestrocel suspensions (*n* = 3) were used to measure their radioactivity with a gamma well counter (2480 WIZARD^2^ automatic gamma counter, PerkinElmer Inc., Waltham, MA). The cells were kept at room temperature before administration.

**Radioactivity from ^51^Cr-labeled suspension**: Specific activity (SA) and coefficient of variance (CV) were calculated using the following formulas:

SA = D/(F × S),

where D = amount of radioactivity from sample, F = cell concentration, and S = sample collection quantity.

CV = SD/A × 100,

where SD = standard deviation of radioactive concentration from suspension, and A = average of radioactive concentration from suspension.

**^51^Cr-labeled nafimestrocel administration**: On P10, the pups received intravenous administration of ^51^Cr-labeled nafimestrocel (1 × 10^6^ cells/body) or HBSS (0.1 mL/body) via the right external jugular vein under anesthesia with isoflurane inhalation (2%–2.5% isoflurane for both induction and maintenance).

**Measurement of radioactivity with a gamma well counter:** Radioactivity from each sample of ^51^Cr-labeled nafimestrocel was measured with a gamma well counter for 1 min. The values were corrected according to the count efficiency and half-life of ^51^Cr (27.7 days). Background radioactivity was measured by using the empty holder. The difference between the radioactivity from each sample and the background radioactivity was determined and used for the analysis.

**Preparation and evaluation of brain samples**: Radioactivity was measured 24, 72, and 168 h after the ^51^Cr-labeled nafimestrocel administration via the right external jugular vein. Rats were sacrificed by intraperitoneal administration of pentobarbital overdose if they were younger than 14 days and by CO_2_ inhalation if they were older than 14 days. The whole bodies were then frozen with carbon dioxide snow. Brains were dissected out and embedded in 4% CMC-Na, and frozen sections (30 µm thickness) were prepared. Coronal sections of the brain were prepared in two planes: one plane contained the striatum and the other contained the hippocampus and optic thalamus. The sections were coated with protective films and used for the bioimaging analyzer system (BAS, FUJIX-BAS2500, Fuji Film, Tokyo, Japan).

**Preparation of standard samples for standard curve production**: Blood samples (*n* = 3) were collected from rats, and the tracer was added to them. The samples were diluted serially to obtain standard samples for standard curve production.

**Calculation of radioactivity concentration**: Brain sections were placed on imaging plates (TYPE BAS SR2040, Fuji Film, Tokyo, Japan) with standard samples. The radioactivity images were obtained from the imaging plates for 24 and 72 h in a lead shield box. Photostimulated luminescence (PSL) was read from the radioactivity images with BAS, and radioluminograms were generated. A standard curve was constructed using the PSL of standard samples. Reading conditions were as follows: resolution = 50 µm, gradation = 256, sensitivity = 10,000, latitude = 4. Regions of interest (ROIs) were set along the periphery of the cerebral parenchyma. Radioactivity in each ROI was calculated as PSL per unit area (PSL/mm^2^). In HI rats, the remaining parenchyma in the ipsilateral hemisphere and that in the contralateral hemisphere was evaluated separately. In sham rats, the whole brain was considered an ROI to detect the smallest amount of radioactivity, and total radioactivity was calculated. Radioactivity concentration was determined as the difference in radioactivity in each tissue and background radioactivity. The average radioactivity concentration obtained from HI rats was compared with the average values in the sham rats for 24–168 h.

***Assessment of injury with T2-weighted MRI***

MRI was performed three days after HI insult (P10) for the assignment of rats to evaluation of safety, behavior, and histopathology. Rat brains were scanned on an MRS 3000/1500 Benchtop System (MR solutions, Guildford, UK) with a 3 T/17 cm cryogen-free superconducting magnet. Anesthesia was induced and maintained with 2.5% isoflurane during scanning. Animals were placed prone on a plastic holder and inserted into a quadrature birdcage imaging coil (20 mm diameter). Eighteen coronal T2-weighted images were taken to confirm each ipsilateral signal change suggesting HI injury, using the following settings: TR = 4500 ms, TE = 68 ms, FOV = 30 mm^2^, slice thickness = 1.0 mm, slice separation gap = 1.1 mm, and average acquisition time = 2 min. Application software (Easyscan, MR Solutions, Guildford, UK) was used for image analysis.

The severity of brain injury was categorized into three grades using the method described by Mikrogeorgiou et al ^35^. : mild (no or little hyperintensity in the parietal cortex), moderate (unilateral hyperintensity occupying the cortex and hippocampus), or severe (unilateral hyperintensity occupying the cortex and hippocampus and extending to the striatum and basal ganglia; Supplemental Figure 1b). Rats with moderate injury were adapted for subsequent experiments.

***Assignment of groups***

We set two time points for cell administration at three and seven days after HI insult (P10 and P14, respectively), to compare the difference between its therapeutic effect. Nafimestrocel was dispensed in HBSS (Thermo Fisher Scientific, Waltham, MA) as the vehicle. The number of injected cells from nafimestrocel was set at 1 × 10^6^ cells/body in HBSS (0.1 mL). HI rats were allocated to three groups: the M3 group (*n* = 9) first received cell administration on P10, followed by an HBSS injection (0.1 mL/body) on P14; the M7 group (*n* = 9) first received an HBSS injection on P10, followed by cell administration on P14; and the vehicle group (*n* = 9) received an HBSS injection at both P10 and P14.

***Preparation of nafimestrocel for administration***

The frozen bag containing nafimestrocel was placed in a heating bucket (37°C), and the thawed cell suspension was transferred to a centrifuge tube. HBSS (15 mL) was added, and the cell suspension was centrifuged at 700 × *g* for 5 min at room temperature. After removal of the supernatant, 30 mL HBSS was added, and the cells were centrifuged again in the same manner. Finally, nafimestrocel was suspended in HBSS at a concentration of 1 × 10^7^ cells/mL and was kept at room temperature before administration.

***Nafimestrocel administration***

The pups were anesthetized with isoflurane inhalation (2%–2.5% isoflurane for both induction and maintenance) and received an intravenous injection of nafimestrocel (1 × 10^6^ cells/0.1 mL) or HBSS (0.1 mL/body) via the right external jugular vein on P10 and P14 according to the group assignment. Immunosuppression was not performed throughout this study.

***Behavioral tests***

Grouping for all behavioral tests and evaluations was done blindly.

***Cylinder test***

The forelimb use preference was calculated as follows: (nonimpaired − impaired)/(nonimpaired + impaired + both) × 100. The trial was performed for three consecutive days. Test duration was set to 10 min/trial. If the frequency of forelimb touch was >10 in 5 min, only the frequency in the first 5 min was used for estimating forelimb use preference. If the frequency of forelimb touch was <10 in 10 min, the data were regarded as inadequate and excluded from analysis. The average frequency for each rat was used for statistical analysis.

***Open-field test***

Each rat was placed in the center of an open-field chamber (width 100 cm × depth 100 cm × height 45 cm), and behavior was recorded for 5 min. The chamber was washed with 70% ethanol between sessions. Measures included distance traveled, rest time, and time spent staying in the center (50 cm^2^ area) and were automatically recorded by a computer using ANY-maze Video Tracking System (Stoelting Co., Wood Dale, IL).

***Water maze test***

A transparent circular platform (12 cm diameter) was placed 2 cm beneath the water surface. The rat was placed at the starting position, and the trial was completed when it reached the platform. The trial was aborted when a rat did not reach the platform within 120 s. The test was performed three times per day. Measures including swimming duration, swimming speed, and distance traveled were recorded with a software. The average values over five days were calculated and used for further analysis.

***Sacrifice for pathological examination***

At ten weeks, rats were sacrificed with isoflurane overdose (inhalation), and their brains and lungs were collected. Brains were weighed before tissue fixation. Both tissues were fixed with 10% neutral buffered formalin, embedded in paraffin, and cut into 5 µm thick sections.

***Injury evaluation***

Brain injury was evaluated using sections at the levels including all aimed regions, namely the cerebral cortex, hippocampus, thalamus, and basal ganglia. A semiquantitative neuropathological scoring system ^44, 45^ (Supplemental Table) was adopted. The enlargement of ventricles was scored from 0–4, with 0 indicating no enlargement and 4 indicating severe enlargement with the disappearance of or small remainder of the hippocampus and cortex. The atrophy of nerve fascicles in the corpus callosum/external capsule and alveus of hippocampus/fimbria of hippocampus were also scored from 0–4, with 0 indicating no atrophy and 4 indicating disappearance of nerve fascicles. Both hemispheres were evaluated. The mean grade in each group was used for analysis.

***Microglial activation assessments***

Microglial activation was assessed using the modified method of Suzuki et al ^33^. In brief, microglia (6-3 Microglia Cell Clone, COS-NMG-6-3C, Cosmo Bio Co., Ltd., Tokyo, Japan) were plated on 24-well plates at a density of 1.1 × 10^4^ cells/cm^2^ (2.0 × 10^4^ cells/well). Medium for Microglia Clone (COS-NMGM, Cosmo Bio Co) containing 33 ng/mL recombinant mouse GM-CSF (415-ML-010/CF, R&D systems, Minneapolis, MN) was used for cell culture. Culture medium was replaced at 24 h and five days after plating the cells.

Nafimestrocel was seeded at a density of 1.8 × 10^4^ cells/cm^2^ (6 × 10^3^ cells/well) on transwell inserts (Boyden chamber: Falcon Cell Culture Insert, Corning Life Sciences, Corning, NY) and cultured in minimum essential medium Eagle, alpha modification (Thermo Fisher Scientific, Waltham, MA) containing 10% fetal bovine serum (Thermo Fisher Scientific, Waltham, MA) and 1 ng/mL basic fibroblast growth factor (Miltenyi Biotec, Bergisch Gladbach, Germany) for 24 h. Subsequently, the nafimestrocel transwell inserts were transferred to the 24-well plates in which microglia were being cultured, 24 h before LPS administration.

The inserts with nafimestrocel were removed six days after starting the culture, and LPS (serotype O55:B5, Sigma-Aldrich, St. Louis, MO) or phosphate-buffered saline was added to microglial cultures at a concentration of 100 ng/mL. Total RNA was extracted from the microglial cultures at 3 and 24 h after adding LPS using RNeasy Mini Kit (Qiagen Inc. Venlo, Netherlands) in accordance with the manufacturer’s protocol. The reverse transcription reaction was performed using 200 ng total RNA using the first-strand cDNA synthesis kit (ReverTra Ace; Toyobo Co., Ltd, Osaka, Japan). Then, quantitative polymerase chain reaction (qPCR) was performed using the LightCycler 96 System (Roche Diagnostics, Indianapolis, IN) and KOD SYBR qPCR Mix (QKD-201, Toyobo Co., Ltd, Osaka, Japan) using the following primers: tumor necrosis factor-α: sense, 5′-GTAGCCCACGTCGTAGCAAAC-3′; antisense, 5′-CTGGCACCACTAGTTGGTTGTC-3′; inducible nitric oxide synthase: sense, 5′-CATGCTACTGGAGGTGGGTG-3′; antisense, 5′-CATTGATCTCCGTGACAGCC-3′; β-actin: sense, 5′-CGTGGGCCGCCCTAGGCACCA-3; antisense, 5′-ACACGCAGCTCATTGTA-3.
