# Supplementary material for: Systemic administration of clinical-grade multilineage-differentiating stress-enduring cells ameliorates hypoxic–ischemic brain injury in neonatal rats
Source: Sci Rep. 2023 Sep 11;13:14958. doi: 10.1038/s41598-023-41026-3 (PMC10495445; doi:10.1038/s41598-023-41026-3)
Supplement: Supplementary file 7 — Supplementary Table 1. [file 41598_2023_41026_MOESM7_ESM.docx]

| Histopathological finding | Grade | Definition |
| --- | --- | --- |
| Enlargement of ventricles |  |  |
|  | 0, none | None |
|  | 1, minimum | Slight enlargment of ventricles, without apparent atrophy of cortex, hippocampus or thalamus |
|  | 2, mild | 30-50% atrophy of hippocampus compared to sham. Slight atrophy of cortex and thalamus |
|  | 3, moderate | More than 70% atrophy of hippocampus and cortex compared to sham. Slight atrophy of thalamus |
|  | 4, severe | Disappearance or small residual of hippocamupus and cortex. About 50% atrophy of thalamus compared to sham. |
|  |  |  |
| Atrophy of nerve fascicle in corpus callosum  and external capsule | | |
|  | 0, none | None |
|  | 1, minimum | Slight thinning of nerve fascicle in corpus callosum and external capsule compared to sham |
|  | 2, mild | 50% thinning of nerve fascicle in corpus callosum and external capsule compared to sham |
|  | 3, moderate | 70% thinning of nerve fascicle in corpus callosum and external capsule compared to sham |
|  | 4, severe | Disappearance of neve fascicle in corpus callosum and external capsule |
|  |  |  |
| Atrophy of nerve fascicle in alveus hippocampus  and fimbria hippocampus | | |
|  | 0, none | None |
|  | 1, minimum | 50-70% thinning of nerve fascicle in alveus and fimbria hippocampus compared to sham |
|  | 2, mild | 80% thinning of nerve fascicle in alveus and fimbria hippocampus compared to sham |
|  | 3, moderate | 90% thinning of nerve fascicle in alveus and fimbria hippocampus compared to sham |
|  | 4, severe | Disappearance of neve fascicle in alveus and fimbria hippocampus |

Supplementary Table. The semiquantitative neuropathological scoring system
